# The Concentration of Essential and Non-Essential Elements of Colostrum, Correlation with Blood Samples of calves, Estimated Daily Intakes and Supplemental Ratio

**DOI:** 10.1007/s12011-026-04994-x

**Published:** 2026-02-05

**Authors:** Fulya Altınok-Yi̇pel, Hüsamettin Eki̇ci̇, Halime Kara, Yaşar Aluç, Mustafa Yi̇pel

**Affiliations:** 1 Faculty of Veterinary Medicine, Department of Veterinary Internal Medicine, Necmettin Erbakan University, Konya, Türkiye; 2 Faculty of Veterinary Medicine, Department of Pharmacology and Toxicology, Kırıkkale University, Kırıkkale, Türkiye; 3 Health Vocational School, Department of Veterinary, Ankara Yıldırım Beyazıt University, Ankara, Türkiye; 4 Kırıkkale University, Scientific and Technological Research Application and Research Center, Kırıkkale, Türkiye; 5 Faculty of Veterinary Medicine, Department of Pharmacology and Toxicology, Hatay Mustafa Kemal University, Hatay, Türkiye

**Keywords:** Calf; essential elements, Non-essential elements, Colostrum, Blood, Correlation, EDI

## Abstract

Feeding a newborn calf in the first hours plays a key role in its survival, health and lifetime performance. Due to the nutritional and biological value of the compounds it contains, colostrum is the calf’s primary food source. Neonatal calves are more susceptible to diseases, and contaminated (such as potentially toxic elements) colostrum may pose health risks. This study aimed to analyse the concentrations and correlations of essential and non-essential elements in colostrum and blood samples, and to assess the potential health risks by estimating the daily intake (EDI). The concentrations in the colostrum were ranked as Sr > Ba > Al > As and Mg > Zn > Fe > Se > Cu > Mo > Cr, whereas in the blood they were ranked as Al > Sr > Ba > As/Ni and Mg > Sn > Fe > Cu > Zn > Se > Cr. Statistically significant positive and negative correlations were identified between the concentrations of elements in colostrum and blood. Although Ba, Cr(III), Cu, Fe, Ni and Sr do not pose a health risk to neonatal calves, Al, As, Cr(IV), Se and Zn may do so according to the EDIs. Colostrum samples were insufficient to meet the daily requirements for Co, Cu, Fe and Mn, and the daily exposure to Se and Zn may pose health risks to calves. Studies involving species-specific parameters and exposure scenarios are needed to adapt the risk assessment method, which could be a key tool in preventive animal health medicine.

## Introduction

The way in which a newborn calf is fed during its first hours of life has a significant impact on its chances of survival, health, and lifetime performance [1, 2]. Neonatal calves are susceptible to disease because they are born agammaglobulinaemic and gain natural immunity by consuming high-quality colostrum at the right time [3]. Due to its rich content of nutritional and biologically active compounds, colostrum is the first food for calf health and is given at a rate of 5 L per day (2.5 L twice daily) or 10% of the calf’s body weight for three days, starting within one hour of birth [4–8]. It is recommended that high-quality colostrum (≥ 50 mg/mL immunoglobulins [IgG]) is given within the first 24 h, as this is when intestinal permeability is at its highest for the absorption of immunoglobulin proteins [9]. Colostrum is composed of immunoglobulins (G1 and G2), epidermal growth factors (insulin-like 1 and 2, and transforming growth factors 1 and 2)., enzymes and inhibitors (e.g. digestive enzymes, proteinases, antioxidants, lipases, lactoperoxidase, phosphatases, lysozyme, cystein protease and trypsin), lactoferrin, cytokines and interleukins (e.g. IL-1, IL-3, IL-4, IL-5, IL-6, IL-8, IL-10, IL-12 and IL-13)., IL-18, IFN-γ, TNF-α and their receptors, nucleosides, nucleotides, leukocytes, exosomes, vitamins (ascorbic acid, thiamine, riboflavin, niacin, pyridoxal, cobalamin, biotin, tocopherol, retinol, β-carotene, cholecalciferol and phylloquinone) and amino acids (essential (lysine, tryptophan, histidine, phenylalanine, threonine, valine, methionine, isoleucine and leucine) and semi-essential (cysteine and tyrosine)). Essential amino acids (threonine, valine, methionine, isoleucine, leucine, phenylalanine, lysine and histidine) and semi-essential amino acids (cysteine and tyrosine), fatty acids (branched, odd-chain, short-chain, medium-chain saturated long-chain saturated, polyunsaturated, monounsaturated, conjugated linoleic acid and trans fatty acids), oligosaccharides (major, neutral and acidic), carbohydrates and macronutrients (calcium, phosphorus, sodium, potassium, sulphur [S] and magnesium), and micro (zinc [Zn], copper [Cu], iron [Fe], etc.) essential elements [5–7, 10]. It is believed that the nutrient content of colostrum obtained during the first milking is high and important for newborn calves [11]. Essential elements such as Cu, Fe, manganese (Mn), Zn and selenium (Se) play an important role in improving immunoglobulin levels, antioxidant status and reproduction in ruminants, and are favoured in commercial formulations used in ruminant nutrition [12, 13]. Although essential elements are necessary for the biological functions of organisms, they can have toxic effects when present in concentrations above the tolerable level [14, 15]. The toxic concentrations (mg kg^− 1^) of non-essential elements in calves were not specified, but were set at 10 mg/kg for molybdenum (Mo) and 2 mg kg^− 1^ for mercury (Hg) in cattle [16]. The concentrations of Mg, Fe, Zn, Cu and Mn in colostrum are high during the first milking, but may decrease after 24 h [11, 17]. Although colostrum is recognised for its nutritional value, our understanding of its composition is still limited [18, 19]. The majority of research on colostrum management has focused on the transfer of maternal IgG to neonates [19]. Compared to mature milk, colostrum contains higher concentrations of essential and potentially toxic metals. Therefore, it is crucial to evaluate the elemental composition of this early milk fraction in order to understand its nutritional and toxicological implications [20]. On the other hand, it may also contain non-essential elements that can pass into the colostrum of neonatal cows due to their exposure to feed, air and water, and which can have toxic effects if the tolerable limits are exceeded [12]. Studies have generally focused on the nutrient content of colostrum. While several of its bioactive and nutritional components have been widely studied, research into its mineral composition remains limited [19]. Although studies have investigated metal concentrations in colostrum samples, research into the concentrations of potentially toxic elements in colostrum and their potential impact on calves is limited [7, 20]. There is limited research into the concentrations of potentially toxic elements in colostrum and their potential impact on calves.

The aim of this study was to: (1) quantify the concentrations of essential elements (Co, Cr, Cu, Fe, Mg, Mn, Mo, Se, Sn, V and Zn) and non-essential elements (Al, As, Ba, Cd, Hg, Ni, Pb and Sr) in colostrum and calf blood samples; (2) assess the correlation between element levels in colostrum and in the corresponding blood samples of calves; (3) evaluate the extent to which colostrum contributes to meeting the recommended daily requirements for essential elements; and (4) estimate the potential health risks in accordance with the estimated daily intake (EDI).

## Materials and Methods

### Sample Collection

Composite colostrum samples (*n* = 24; 10 ml each) were collected from 24 different newly calved Holstein cows (once sample from each) at the first milking after calving (6 h ± 30 min) on the same farm. The samples were stored in 10 ml tubes at −80 °C until elemental analysis. Each calf was fed a total of 5 L of colostrum of its own dam twice a day for three days. Blood samples were collected from the calves 24 h after colostrum feeding. Blood samples (*n* = 24) were collected from the jugular vein of the calves into tubes with and without anticoagulant (6 ml). The samples were then centrifuged at 3000 rpm for 10 min, after which the serum samples were obtained and stored at −80 °C until elemental analysis. This study was approved by the Kırıkkale University Animal Local Ethics Committee (334270).

### Element Analysis

For the exraction; HNO₃ (10 mL, 65%) to the colostrum samples (4 mL), H₂O₂ (1 mL) and HNO₃ (5 mL, 65%) to the blood samples (1 mL) were added and digested by a microwave system (CEM Mars 6, Matthews, NC, USA) under the operating conditions given in Table 1. The concentrations of essential (Co Cr, Cu, Fe, Mg, Mn, Mo, Se, Sn, V, and Zn) and non-essential (Al, As, Ba, Cd, Hg, Ni, Pb, and Sr) elements in extracts were measured by inductively coupled plasma mass spectrometry (ICP-OES) (SpectroBlue, Germany) under the operating conditions given in Table 2 [21].

**Table 1:** Microwave operating conditions for non-essential and essential element analyzes

Power (W)	Ramp time (min)	Hold time (min)	Temperature (°C)
290–1800	20:00	15:00	200

**Table 2:** ICP-OES operating conditions for non-essential and essential element analyses

Plasma power (W)	Pump speed (rpm)	Coolant flow (L min^− 1^)	Auxiliary flow (L min^− 1^)	Nebulizer flow (L min^− 1^)	Number of replicates	Integration time (s)	Sample uptake rate (µL min^− 1^)
1435	30	13	0.80	0.75	3	3	0.3

The quantitative measurement of elements was evaluated using external calibration by drawing calibration curves from 13 points between 0 and 2 ppm. The limit of detection (LOD), limit of quantitation (LOQ), recoveries and correlation coefficients (r²) of the validation are given in Table 3.

**Table 3:** The validation results of non-essential and essential element analyses

Elements	Limit of Detection (µg kg^− 1^)	Limit of Quantitation (µg kg^− 1^)	Recovery (%)	Correlation Coefficient (*r*^2^)
Al	0.84	2.55	105.6	0.999
As	3.68	11.15	88.9	0.999
Ba	0.15	0.45	89.3	0.999
Cd	0.15	0.45	96.1	0.999
Co	0.60	1.82	88.3	0.999
Cr	0.48	1.45	95.4	0.999
Cu	0.93	2.82	107.8	0.999
Fe	1.84	5.58	77.5	0.999
Hg	1.24	3.76	98.9	0.999
Mg	0.74	2.24	90.0	0.999
Mn	0.91	2.76	86.3	0.999
Mo	2.11	6.39	93.5	0.999
Ni	2.55	7.73	76.5	0.999
Pb	2.23	6.76	80.9	0.998
Se	5.33	16.2	96.9	0.999
Sn	8.31	25.2	94.6	0.999
Sr	0.32	0.97	86.8	0.999
V	1.66	5.03	101.4	0.999
Zn	0.27	0.82	79.4	0.999

### Estimation of the Element Daily Intake

The estimated daily intake (EDI) of elements for calves (both male and female) related to colostrum consumption was calculated according to the following formula [4, 22, 23].

$$\:\mathrm{E}\mathrm{D}\mathrm{I}=\frac{\mathrm{C}\mathrm{o}\mathrm{l}\mathrm{o}\mathrm{s}\mathrm{t}\mathrm{r}\mathrm{u}\mathrm{m}\:\mathrm{i}\mathrm{n}\mathrm{t}\mathrm{a}\mathrm{k}\mathrm{e}\:\left({5L\:calf\:day}^{-1}\right)\times\:\mathrm{E}\mathrm{l}\mathrm{e}\mathrm{m}\mathrm{e}\mathrm{n}\mathrm{t}\:\mathrm{c}\mathrm{o}\mathrm{n}\mathrm{c}\mathrm{e}\mathrm{n}\mathrm{t}\mathrm{r}\mathrm{a}\mathrm{t}\mathrm{i}\mathrm{o}\mathrm{n}\:\left({mg\:L}^{-1}\right)}{\mathrm{A}\mathrm{v}\mathrm{e}\mathrm{r}\mathrm{a}\mathrm{g}\mathrm{e}\:\mathrm{b}\mathrm{o}\mathrm{d}\mathrm{y}\:\mathrm{w}\mathrm{e}\mathrm{i}\mathrm{g}\mathrm{h}\mathrm{t}\:\mathrm{o}\mathrm{f}\:\mathrm{a}\:\mathrm{c}\mathrm{a}\mathrm{l}\mathrm{f}\:\left(\mathrm{m}\mathrm{a}\mathrm{l}\mathrm{e}\:40.07\:\mathrm{k}\mathrm{g}\:\mathrm{a}\mathrm{n}\mathrm{d}\:\mathrm{f}\mathrm{e}\mathrm{m}\mathrm{a}\mathrm{l}\mathrm{e}\:28.06\:\mathrm{k}\mathrm{g}\right)}$$

### Statistical Analysis

The Shapiro-Wilk and Spearman’s correlation coefficient (r) tests were performed using the statistical programme (SPSS 27.0, IBM, USA). Differences at the 0.05 level of significance were defined as significant.

## Results

The concentrations (mg L^− 1^) of non-essential (Al, As, Ba, Cd, Hg, Ni, Pb, Sr) and essential (Co Cr, Cu, Fe, Mg, Mn, Mo, Se, Sn, V, Zn) elements in colostrum and blood samples (*n* = 24) are presented in Tables 4 and 5.

**Table 4:** The concentrations (mg L^−1^) of non-essential and essential elements in colostrum and blood samples (*n* = 24)

Element		Arithmetic mean	Standard error	Standard deviation	Median	Geometric mean	Minimum	Maximum
Non-Essential	**Al**	0.09	0.06	0.28	*< LOD*	*< LOD*	*< LOD*	1.35
	**As**	0.01	0.00	0.00	0.01	*< LOD*	*< LOD*	0.02
	**Ba**	0.15	0.01	0.06	0.15	0.14	0.06	0.31
	**Cd**	*< LOD*	-	-	*< LOD*	*< LOD*	*< LOD*	*< LOD*
	**Hg**	*< LOD*	0.00	0.02	*< LOD*	*< LOD*	*< LOD*	0.08
	**Ni**	*< LOD*	-	-	*< LOD*	*< LOD*	*< LOD*	*< LOD*
	**Pb**	*< LOD*	-	-	*< LOD*	*< LOD*	*< LOD*	*< LOD*
	**Sr**	1.74	0.10	0.51	1.80	1.67	0.82	2.64
Essential	**Co**	*< LOD*	-	-	*< LOD*	*< LOD*	*< LOD*	*< LOD*
	**Cr**	0.02	0.00	0.01	0.02	0.02	0.01	0.04
	**Cu**	0.21	0.02	0.09	0.19	0.20	0.07	0.44
	**Fe**	1.47	0.17	0.85	1.18	1.28	0.55	4.24
	**Mg**	302.6	13.50	66.15	303.11	295.3	162.3	444.9
	**Mn**	*< LOD*	-	-	*< LOD*	*< LOD*	*< LOD*	*< LOD*
	**Mo**	0.03	0.00	0.02	0.03	< LOD	< LOD	0.10
	**Se**	0.25	0.01	0.03	0.25	0.25	0.20	0.33
	**Sn**	*< LOD*	0.00	0.01	*< LOD*	*< LOD*	*< LOD*	0.05
	**V**	*< LOD*	0.00	0.01	*< LOD*	*< LOD*	*< LOD*	0.04
	**Zn**	16.85	1.33	6.53	17.90	15.29	4.37	26.86

**Table 5:** Essential element concentration of calf blood samples (mg L^−1^) (*n* = 24)

Element		Arithmetic mean	Standard error	Standard deviation	Median	Geometric mean	Minimum	Maximum
Non-Essential	**Al**	1.07	0.06	0.31	1.09	1.03	0.61	1.81
	**As**	0.01	0.00	0.00	0.01	0.01	*< LOD*	0.01
	**Ba**	0.02	0.00	0.00	0.02	0.02	0.01	0.02
	**Cd**	*< LOD*	-	-	*< LOD*	*< LOD*	*< LOD*	*< LOD*
	**Hg**	*< LOD*	-	-	*< LOD*	*< LOD*	*< LOD*	*< LOD*
	**Ni**	0.01	0.00	0.01	0.01	*< LOD*	*< LOD*	0.04
	**Pb**	*< LOD*	-	-	*< LOD*	*< LOD*	*< LOD*	0.01
	**Sr**	0.19	0.01	0.05	0.20	0.19	0.12	0.35
Essential	**Co**	*< LOD*	-	-	*< LOD*	*< LOD*	*< LOD*	0.01
	**Cr**	0.04	0.01	0.04	0.03	0.04	0.03	0.24
	**Cu**	0.64	0.03	0.13	0.64	0.63	0.44	0.96
	**Fe**	1.87	0.19	0.92	1.78	1.70	0.71	5.37
	**Mg**	15.59	0.75	3.69	14.82	15.23	10.75	27.51
	**Mn**	*< LOD*	-	-	*< LOD*	*< LOD*	*< LOD*	*< LOD*
	**Mo**	*< LOD*	-	-	*< LOD*	*< LOD*	*< LOD*	*< LOD*
	**Se**	0.17	0.00	0.02	0.18	0.17	0.14	0.19
	**Sn**	5.36	0.20	0.96	5.46	5.24	1.83	7.46
	**V**	*< LOD*	-	-	*< LOD*	*< LOD*	*< LOD*	*< LOD*
	**Zn**	0.45	0.03	0.14	0.50	0.43	0.13	0.63

The concentrations of Ba, Sr, Cr, Cu, Fe, Mg, Se and Zn were above the LOD in all colostrum and blood samples. Al concentrations were above the LOD in five (20.8%) colostrum samples and all blood samples. As concentrations were above the LOD in 23 (95.8%) of the colostrum samples and all of the blood samples. Hg concentrations were above the LOD in one (4.2%) colostrum sample and below the LOD in all blood samples. Lead (Pb) concentrations were below the LOD in all colostrum samples and above the LOD in one (4.2%) blood sample. The Cobalt (Co) concentrations were above the LOD in three (12.5%) colostrum samples and one (4.2%) blood sample. Mn concentrations were below the LOD in all colostrum and blood samples. Mo concentrations were above the LOD in 20 (83.3%) of the colostrum samples and below the LOD in all of the blood samples. Ni concentrations were below the LOD in all colostrum samples, while 22 (91.7%) blood samples were above the LOD. Sn concentrations were above the LOD in three (12.5%) colostrum samples and all blood samples. V concentrations were above the LOD in five (20.8%) colostrum samples and below the LOD in all blood samples.

The concentrations of non-essential and essential elements in colostrum were ranked as Sr > Ba > Al > As and Mg > Zn > Fe > Se > Cu > Mo > Cr, and as Al > Sr > Ba > As/Ni and Mg > Sn > Fe > Cu > Zn > Se > Cr in blood samples of calves.

The highest and lowest measured values of non-essential and essential elements in the colostrum were as follows Al (*< LOD*−1.35), As (*< LOD*−0.02), Ba (0.06–0.31), Hg (*< LOD*−0.08), Sr (0.82–2.64 and Cr (0.01–0.04), Cu (0.07–0.44), Fe (0.55–4.24), Mg (162.3–444.9.3.9), Mo (< LOD- 0.10), Se (0.20–0.33), Sn (*< LOD*- 0.05), V (*< LOD*- 0.04), Zn (4.37–26.86). The highest and lowest measured values of both non-essential and essential elements in blood were as follows: Al (0.61–1.81), As (*< LOD*−0.01), Ba (0.01–0.02), Ni (*< LOD*−0.04), Pb (*< LOD*−0.01), Sr (0.12–0.35) and Co (*< LOD*−0.01), Cr (0.03–0.24), Cu (0.44–0.96), Fe (0.71–5.37), Mg (10.75–27.51), Se (0.14–0.19), Sn (1.83–7.46), Zn (0.13–0.63). Ba, Sr, Mg, Mo, Se, and Zn concentrations were higher in the colostrum than in the blood.

The correlation levels between the concentrations of non-essential and essential elements in colostrum samples and blood samples of calves are presented in Table 6.

**Table 6:** The correlation levels between non-essential and essential element concentrations between colostrum samples and blood samples of calves

Blood Colostrum		Non-Essential				Essential							
		Al	As	Ba	Sr	Co	Cr	Cu	Fe	Mg	Se	Sn	Zn
Non-Essential	**Al**	0.185	−0.033	−0.030	−0.081	−0.067	−0.075	0.032	0.066	−0.221	0.124	−0.080	−0.078
	**As**	0.455^*^	0.122	0.235	−0.125	−0.030	0.665^**^	−0.414^*^	0.226	−0.473^*^	0.386	0.835^**^	−0.050
	**Ba**	0.172	0.127	0.055	0.086	−0.362	0.312	0.236	0.088	0.318	−0.198	−0.168	−0.168
	**Sr**	0.260	0.177	0.068	0.036	−0.386	0.170	0.105	0.080	0.195	−0.295	−0.100	−0.097
Essential	**Co**	0.155	−0.074	−0.193	−0.352	−0.072	−0.068	−0.300	0.371	−0.017	−0.135	0.165	−0.515^**^
	**Cr**	0.498^*^	0.082	0.150	−0.025	−0.331	0.315	−0.209	0.213	0.108	−0.216	0.263	−0.234
	**Cu**	0.308	−0.174	0.044	−0.283	−0.229	0.276	−0.116	0.065	0.042	−0.137	0.312	−0.422^*^
	**Fe**	−0.140	−0.239	−0.130	0.065	−0.034	−0.152	−0.047	−0.159	−0.341	0.291	0.210	−0.353
	**Mg**	0.443^*^	−0.087	0.145	−0.176	−0.452^*^	0.087	−0.011	0.156	0.026	−0.290	0.200	−0.207
	**Mo**	0.296	−0.097	−0.005	−0.103	−0.285	0.005	−0.014	−0.006	0.224	−0.443^*^	−0.040	−0.302
	**Se**	−0.421^*^	−0.475^*^	−0.146	−0.020	0.016	−0.315	0.111	0.119	0.010	0.186	0.042	−0.031
	**Sn**	0.151	0.071	−0.333	−0.303	−0.072	−0.092	−0.489^*^	0.203	−0.200	−0.038	−0.074	−0.492^*^
	**Zn**	0.453^*^	0.027	0.290	−0.060	−0.370	0.235	0.169	0.247	0.223	−0.291	0.009	−0.173

*(p < 0.05), **(p < 0.01)

Statistically significant positive correlations were identified between colostrum As and blood Al (*p* < 0.05), Cr, and Sn (*p* < 0.01), whereas negative correlations were noted with blood Cu and Mg (*p* < 0.05). Furthermore, there was a significant positive correlation between colostrum Cr and blood Al (*p* < 0.05). A statistically significant negative association was observed between colostrum Co and blood Zn (*p* < 0.01), and similarly, as well as between colostrum Cu and blood Zn (*p* < 0.05). Additionally, colostrum Mg showed a positive correlation with blood Al (*p* < 0.05), and a negative correlation with blood Co (*p* < 0.05). A significant negative correlation was also detected between the concentration of Mo in colostrum and the concentration of Se in blood (*p* < 0.05). A negative correlation was found between colostrum Se and both blood Al and As (*p* < 0.05). Furthermore, there were significant negative correlations between colostrum Sn and blood Cu and Zn concentrations (*p* < 0.05). Finally, a positive correlation was identified between the concentration of Zn in the colostrum and the concentration of Al in the blood (*p* < 0.05).

The estimated daily intake (EDI) of both non-essential and essential elements for female and male calves are presented in Table 7.

**Table 7:** EDI (mg kg day^− 1^), reference dose for oral exposure (RfD) (mg kg day^− 1^) and critical effects of non-essential and essential for calves [24–26]

Element		EDI		RfD	Affected Systems and/or Critical Effects
		Female	Male		
Non-Essential	Al	1.2 × 10^− 2^	1.1 × 10^− 2^	4.0 × 10^− 4^	Body weight and clinical parameters
	As	1.3 × 10^− 3^	1.2 × 10^− 3^	6.0 × 10^− 5^	Endocrine, cardiovascular. ischemic heart disease and type 2 diabetes
	Ba	2.0 × 10^− 2^	1.9 × 10^− 2^	2.0 × 10^− 1^	Urinary, nephropathy
	Ni	1.3 × 10^− 3^	1.2 × 10^− 3^	2.0 × 10^− 2^	Decreased body and organ weights
	Sr	2.3 × 10^− 1^	2.2 × 10^− 1^	6.0 × 10^− 1^	Musculoskeletal, rachitic bone
Essential	Cr	2.6 × 10^− 3^	2.5 × 10^− 3^	1.5 (CrIII) 9.0 × 10^− 4^ (CrIV)	No effects observed Gastrointestinal
	Cu	2.8 × 10^− 2^	2.6 × 10^− 2^	4.0 × 10^− 2^	-
	Fe	1.9 × 10^− 1^	1.8 × 10^− 1^	7.0 × 10^− 1^	Gastrointestinal
	Se	3.3 × 10^− 2^	3.1 × 10^− 2^	5.0 × 10^− 3^	Nervous, hematologic, dermal
	Zn	2.2	2.1	3.0 × 10^− 1^	Immune, hematologic, decreases in erythrocyte Cu, Zn-superoxide dismutase activity

The elements were ranked in descending order of concentration as follows: Sr > Ba > Al > Ni = As for non-essential elements, and Zn > Fe > Se > Cu > Cr for essential elements. As the EDI values for Ba, Cr(III), Cu, Fe, Ni and Sr are lower than the reference doses (RfDs), daily exposure to these elements does not pose a significant non-carcinogenic health risk to neonatal calves. However, the EDIs for Al, As, Cr (IV), Se, and Zn were higher than the reference doses (RfDs), suggesting that daily exposure to these substances could pose a health risk to neonatal calves.

Although there is no data on the daily essential element requirements of calves during the first three days of consuming colostrum, the recommended concentrations of milk replacer feed and the rate at which these concentrations are achieved, based on the averages in our colostrum samples, are presented in Table 8.

**Table 8:** The recommended daily concentrations of essential elements (mg 45 kg Bw DM day^−1^) for calf milk replacers and the colostrum supplementation ratio (%), as determined in this study

Essential Elements	Recommended Concentrations (NRC, 2001)	Concentration of daily essential element intake by colostrum in this study (mg day^− 1^)	Daily Supplemental Ratio (%)
Co	0.11	*< LOD*	0
Cu	10	1.05	10.5
Fe	100	7.35	7.4
Mn	40	*< LOD*	0
Se	0.3	1.25	416.7
Zn	40	84.25	210.6

According to the results, the ratios of colostrum to the recommended concentrations of the essential elements studied were ranked as Se > Zn > Cu > Fe. The Co and Mn concentrations in the colostrum samples were below the LOD in this study.

## Discussion

Essential elements such as Cu, Mn, Se, and Zn are critical for the biological activity and metabolism of newborn calves, and they affect the quality of colostrum. Therefore, they may indicate the quality of colostrum and its importance for healthy nutrition [12, 27]. In this study, in addition to the essential elements most frequently included in the studies (Se, Co, Cu, Fe, Zn), Co, Cr, Mo, Se, Sn, V were also analysed [8, 11, 18, 27–29]. It has been reported that the content of the minerals (Ca, Mg, Na, Zn, Fe, Cu, Mn) in colostrum obtained from the first milking is higher than in subsequent milkings [11, 29]. The comparison of the concentrations of analysed essential and non-essential elements in colostrum, as presented in this study and in the literature, are presented in Table 9.

**Table 9:** Concentrations (ppm) of elements in colostrum in this study and as reported in the literature for different countries

Element	This Study	Valldecabres and Silva-del-Río, [11]	Aydogdu and Guzelbektes, [29]	Mohrekesh et al., [30]	Niwińska, and Andrzejewski, [31]	Fantuz et al., [32]	Kehoe et al., [18]	Pavlata et al., [27]	Tammam et al., [28]
Cd	*< LOD*	**-**	0.02	**-**	**-**	**-**	**-**	**-**	0.0006^*^
Cr	0.02	-	0.09	-	-	-	-	-	-
Cu	0.21	0.21	0.79	-	-	0.06	0.34	0.34	-
Hg	*< LOD*	-	-	-	-	-	-	-	0.0003^*^
Fe	1.47	0.70	3.19	-	-	0.21	5.33	-	0.58
Mg	302.6	326.66	455.23	-	-	104.0	733.2	-	-
Mn	*< LOD*	-	0.25	-	-	0.03	0.10	-	0.04
Mo	0.03	-	-	-	-	0.07	-	-	-
Se	0.25	-	-	0.12	0.23–0.39	0.03	-	0.04	0.0023^*^
Zn	16.85	17.79	29.44	-	-	3.44	38.10	27.25	-

The units of element concentrations in the studies have been converted to ppm and rounded to two decimal places

* The numbers has not been able to rounded to two decimal places because are very low

These differences in colostrum composition may be related to the cows’ diet, the season, the breed, the number of births, the milking interval, and the variables in the studies [11].

In the present study, the average Cr concentration (mg L^− 1^) in colostrum was 0.02, Mn, Hg and Cd average concentrations were below the detection limits. Aydoğdu and Güzelbektaş [29] reported Cr concentrations in colostrum that were five times higher than this study, as well as concentrations above thedetection limits for Mn and Cd. Additionally, the concentrations of Mn, Cd and Hg in this study’s samples were below the detection limit, whereas Tammam et al. [28] detected concentrations of 0.0379, 0.0006 and 0.0003 mg L⁻¹, respectively. Even at low concentrations, non-essential elements can have toxic effects, exceeding tolerable limits and being associated with many diseases. They may also interact with essential elements, thereby inhibiting their beneficial effects [15, 33]. The accumulation of toxic metals in the tissues and/or organs is important not only for animal health but also for humans due to comsumption of products [33, 34]. In addition, the presence of toxic non-essential elements directly related to environmental pollution such as As, Cd, Hg in cattle and are also used as an indicator [7, 33, 35]. Se concentrations (ppm) in colostrum (0.14) samples of supplemented dams and in bloods samples of calves (in first 24 h) (0.12) reported by Mohrekesh et al. (2018) were lower than in this study (0.25 and 0.17) [30]. Colostrum concentrations (mg kg^− 1^) reported by Niwińska and Andrzejewski are higher (0.23–0.39) than in this study (0.25 and 0.17), while blood concentrations (0.03–0.04) are lower [31].

It was reported that the concentrations (µmol L⁻¹) of Zn, Cu and Se in calves’ blood samples after colostral feeding were 26.40, 7.53 and 0.93, respectively [27]. Pavlata et al. [27] reported that the concentrations (µmol L^− 1^) of Zn, Cu and Se in calves’ blood samples after colostral feeding were 26.40, 7.53, and 0.93, respectively [27]. However, compared to those reported by Pavlata et al. [27], the concentrations (mg L⁻¹) of Cu (0.63) and Se (0.17) were higher; the Zn concentration (0.45) was markedly lower in our study. According to Tabrizi et al. [37], the serum Cu concentration in newborn calves was reported to be 99.5 µg dl^− 1^ 24 h after colostrum consumption, emphasising the importance of early colostrum consumption in increasing Cu concentrations in calves [37]. In this study, the serum Cu concentrations (mg L^− 1^) of calves were recorded as being in the range of 0.44–0.96, which is consistent with previous studies.

It has been reported that the concentrations of colostrum Cd, Se, Cu and Mn are higher than those of normal milk, in particular, the concentration of Zn in colostrum is three times higher than in milk. It has been suggested that this may be due to the mineral supplements (e.g. salt blocks) that are given during the last months of pregnancy. It is therefore recommended that you pay attention to this [12]. Studies conducted in Türkiye and other countries, the average concentration of Zn in milk was higher compare to the average concentration of colostrum in this study [36, 38, 39]. Pavlata et al. [27] reported that Zn may accumulate as a result of exposure during intrauterine development in calves as well as its tendency to accumulate in colostrum [27].The higher Cu and Se concentrations in blood than colostrum in this study, supports that colostrum may be the source [7, 18, 40]. In this study, despite colostrum being the only food source for neonates, the average concentrations of Al, Ni and Sn were low or below the detection limits in colostrum, the blood concentrations of calves were above the detection limits, suggesting possible placental transmission or environmental contamination (shelter material, water, etc.) [7]. Onversely, elements like Zn and Sr exhibited high concentrations in colostrum but limited reflection in blood, may indicating restricted absorption or postnatal regulatory control in our study.

The prevalence of non-essential elements (As, Cd, Ni, Cu, etc.) in dairy products has been reported, and As, Cd, Ni, and Hg were at low concentrations or below detection limits in both colostrum and calf blood in our study [7]. It has been reported that Ni, Cd and Hg concentrations (mg L^− 1^) (833, 1 and 60, respectively) were high in milk samples, especially in areas with intensive mining activities. The concentrations in this study indicate that environmental pollution in the region where the study was conducted may be limited. Additionally, Pb concentrations in both the colostrum and the calves’ blood were below the detection limit. This suggests that the calves were exposed to low levels of Pb and were in a safer environment in terms of Pb exposure. The obtained data indicate that regional, environmental and geological factors may determine the concentrations of elements passed to newborns through colostrum [7].

When the results of the study by Lichtmannsperger et al. were compared with those of this study, it was found that the concentrations of As and Ba were similar, whereas the concentration of Mo was low [7]. Although the concentrations of Mo were within the physiological limits in both studies, the observed differences in the concentrations of this and other elements can be explained by variables such as environmental factors, diet, and soil structure. It has been reported that high Mo levels in pastures lead to decreased milk yield and health problems in animals [41]. It should be taken into consideration that the level of Mo in colostrum may be sensitive to regional differences and monitoring may be important for calf health.

The results of this study support the view that the composition of colostrum, particularly the concentration of microelements, can vary depending on the geographical region, farm management practices, and feeding regimes. Therefore, colostrum should not only be considered in terms of immunoglobulins [19].

It has been demonstrated that the elements used in animal nutrition interact with each other (e.g. Zn-Fe, Cu-Fe, Cu-S and Cu-Mo), either increasing or decreasing their own absorption and that of the other elements [16, 42]. In line with the correlations between essential and non-essential elements in colostrum and calf blood (Table 6), this study observed that the absorption of Cu and Mg in calves may be affected if arsenic (As) levels in colostrum are high. High levels of S and Mo can interact negatively with Cu and other minerals (Se, Mn, Zn) by forming thiomolybdates in the rumen, which can seriously reduce Cu absorption [42–45]. Cu interacts antagonistically with elements such as Fe, Mo and S [45, 46]. Although there is no literature on the direct effects of As on Cu and Mg, it is known that As disrupts rumen flora and may inhibit Cu absorption with a similar antaogonistic effect [47]. Further studies are needed to explain the relationship between As and these elements. In addition, in this study, a negative correlation was found between colostrum Mo concentration and calf blood Se concentration in accordance with the literature [43–45]. Cu is known to inhibit Zn uptake in the intestines [48, 49]. The findings of this study suggest that Co, Cu and Sn in colostrum may affect the elimination of Zn in calves. Another finding that supports the negative correlation between Co and Zn is that high concentration of Zn supplementation lead to an imbalance in Co intake in ruminants, thereby preventing the conversion of Co to vitamin B_12_ [49, 50]. Another positive correlation was observed in this study between the As in the colostrum samples and the Al, Cr and Sn in the blood samples of calves. According to these data, an increase in As may lead to an increase in the absorption of Al, Cr and Sn. Colostrum contains both essential and non-essential elements, which may interact with each other to increase their effects [7, 28]. Although not statistically significant, our study observed a positive correlation between the colostrum and calf blood Se concentration, in parallel by Juniper et al. [51]. They also indicated that the correlation between the dam and calf may vary depending on how the Se supplement is metabolised [31, 51].Therefore, studies are needed on the concentrations of colostrum elements, how they are absorbed by calves, and their potential for interacting with each other.

In order to accurately estimate the potential non-carcinogenic health effects of chemical contaminants in breast milk and formula for newborns and children, the risk assessment approach based on RfD has been developed in recent years [52]. However, risk assessment adaptation studies for adults in the field of animal health are limited and insufficient for babies (newborns, neonates, infants or young animals) [53, 54]. Studies on the concentration of elements and risk assessment have focused on cow’s milk and humans who consume it. There are no studies on neonatal calves and colostrum, despite the fact that they are in a period of disease predisposition [39, 55–57]. Although there are no risk assessment studies related to colostrum consumption by calves, studies have identified elemental risks associated with the consumption of breast milk or formula by infants [58–61]. While Cu and Fe absorption is 60% in the first weeks of life (first 4 weeks), this rate decreases as the rumen develops (Cu: 1–5%, Fe: 2%). Considering the insufficient Fe content of milk, supplementation of these elements is recommended, especially for calves that continue to be fed whole milk. The recommended supplementary concentrations of essential elements in calf feed decrease as their lifespan increases. Colostrum has a higher content of essential minerals and vitamins than milk and has an important place as a source of nutrition as well as protection against diseases. Therefore, feeding whole milk instead of colostrum may result in deficiencies of essential elements. Continuing to feed calves fed adequate colostrum with milk and feed containing the required levels of vitamins and minerals can complete maternal deficiencies [16].

In terms of the daily essential element requirements of calves, it was determined that colostrum samples were insufficient in terms of Co and Mn, i.e., it did not contribute to calf nutrition. They were also low in terms of copper (Cu) and iron (Fe), contributing only approximately 10% and 7%, respectively. As the concentrations of Co, Cu, Fe and Mn were far below the recommended levels, it is thought that studies should be carried out on the necessity of classifying colostrum in terms of these elements.

It is important to feed the mother according to her metabolic needs during the dry period, as this ensures their health as well as calves through the quality of their colostrum. After vitamins (24%, *n* = 70), the second most widely studied group of nutritional supplements in the dry period was essential elements (22%), the most widely studied of which was Se (7.1%), followed by combinations of elements such as Co, Cu and Zn (Serhal et al., 2025). Studies have also shown that the proportion of selenium (Se) in the colostrum or blood of calves can vary depending on the form of selenium (Se) supplemented to cows [31, 51].

The determination of Se and Zn concentrations above the daily requirement (approximately four and two times, respectively) supports the findings that these levels may lead to health problems. In this study, daily Se intake (mg day^− 1^) was higher (1.25) than that reported by Niwińska and Andrzejewski at first 24 h (0.28–0.54) from colostrum [31].

The EDI is calculated by dividing the total daily intake of elements (daily colostrum intake multiplied by element concentration) by the average body weight of the consumer groups (male and female calves). If an EDI value that is greater than the RfD means that consuming contaminated food may pose a health risk. There is a lack of up-to-date reference values for the daily concentrations of essential elements in calves. Taking RfD values into account when updating values will help to prevent non-carcinogenic risks. The reason is that RfD values are calculated based on toxic dose studies conducted on animals, as well as the use of safety coefficients to account for variability between species and individuals and data uncertainties [62]. Additionally, body weight is an important factor when considering daily requirements and toxic doses, and so it should be included in calculations, bearing in mind that it can vary according to gender and breed.

Due to the increased metabolic requirements of the elements before calving and during lactation, the concentration of these elements in the diet of pregnant and lactating dams is increased or supplemented. Therefore, in terms of neonatal calf health, may it is a critical situation due to physiological differences (such as increased intestinal permeability) that may cause the ratio of colostrum intake to exceed tolerable limits. The dietary concentrations of Se and Zn in the pregnant, dry, lactation and post-colostrum periods should be dosed according to the metabolism of the calves/dams and the toxicodynamic/kinetic parameters of these elements.

## Conclusions

This study investigated the concentrations of essential and non-essential elements in calf blood and fed colostrum samples, as well as the relationship between the colostrum and blood concentrations and the dynamic interactions between elements. The study also explained the daily essential element supplementation rates through estimated intake amounts, as well as the potential non-carcinogenic health risks associated with exposure. We drew attention to the lack of data on the elemental profile of colostrum during the neonatal stage, which is an important period in cattle farming in terms of both economics and animal health, as high mortality rates can occur. Although data on the nutrient content and elemental levels of colostrum are available, data on the proportion of essential elements that meet calves’ daily requirements and the risks associated with exposure to essential and non-essential elements are scarce. While some elements are physiologically essential, all are potentially toxic above tolerable concentrations. As the digestive systems of neonatal calves are not fully developed and colostrum can contain higher concentrations of elements, it is critically important to keep elemental concentrations within ideal limits. For these reasons, more comprehensive research is needed on the interaction between nutrients, the correlation between blood profiles and health risk potentials, and measuring their concentrations, in order to fully understand the nutritional/toxicological potential of nutrients with possible interactions between elements, which may lead to a decrease/increase in their levels and thus to deficiency/excess or exposure. This study also aimed to stimulate the development of study designs by providing a better characterisation of the daily intake/exposure of neonatal calves to elements in colostrum, as well as the rate at which their daily needs are met and the potential health risks involved. A comparison of reference values with the essential and non-essential elements to which colostrum-fed calves are exposed may be useful in raising awareness of potential health risks at this early stage of life. In order to distinguish between exposure (which could lead to toxic effects) and the daily supplemental ratio (necessary for sustainable health) of elements in neonates, the link between the following parameters needs to be clarified by future conceptualisations: (1) the pharmacokinetics/dynamics of the elemental form (in the diet/supplements of dams and colostrum-fed calves); (2) interactions between elements; and (3) correlations between concentrations in colostrum and calf blood. Meanwhile, for studies like this, which is in the adaptation phase, scenarios including reference values, species-specific parameters 0(such as RfD, absorption and metabolisation functions, breed, breeding type, acute/chronic exposure, other feed contaminants, physiological/health status, etc.) may be useful. Additionally, including analysis of the element concentrations of the dam’s blood, feed, and water in future studies will contribute to the proper interpretation of the findings, concerning the origin and variability of the elements. The reference limits for essential and toxic elements in colostrum in erms of calf nutrition are insufficient. The study revealed that discrepancies may exist between EDI values and daily requirement levels. Therefore, the concentrations of daily essential element requirements should be revised based on RfD values.

## Data Availability

No datasets were generated or analysed during the current study.
